# The effect of insulin administration on c-peptide in critically ill patients with type 2 diabetes

**DOI:** 10.1186/s13613-017-0274-5

**Published:** 2017-05-12

**Authors:** Marco Crisman, Luca Lucchetta, Nora Luethi, Luca Cioccari, Que Lam, Glenn M. Eastwood, Rinaldo Bellomo, Johan Mårtensson

**Affiliations:** 1 Department of Intensive Care, Austin Hospital, The University of Melbourne, 145 Studley Rd, Heidelberg, Melbourne, VIC 3084 Australia; 2 Department of Anesthesia and Intensive Care, Azienda Ospedaliero-Universitaria “Ospedali Riuniti”, Trieste, Italy; 3 Department of Pathology, Austin Health, Melbourne, VIC Australia; 4 Department of Epidemiology and Preventive Medicine, Australian and New Zealand Intensive Care Research Centre, Monash University, Melbourne, VIC Australia; 5 Section of Anaesthesia and Intensive Care Medicine, Department of Physiology and Pharmacology, Karolinska Institutet, 171 77 Stockholm, Sweden

**Keywords:** C-peptide, Blood glucose, Insulin, Diabetes mellitus, Critical care, beta-cell

## Abstract

**Background:**

In critically ill patients with permissive hyperglycemia, it is uncertain whether exogenous insulin administration suppresses or enhances c-peptide secretion (a marker of pancreatic beta-cell response). We aimed to explore this effect in patients with type 2 diabetes.

**Methods:**

We prospectively enrolled a cohort of 45 critically ill patients with type 2 diabetes managed according to a liberal glucose protocol (target blood glucose 10–14 mmol/l). We recorded the administration of insulin and oral hypoglycemic agents and measured plasma c-peptide as surrogate marker of endogenous insulin secretion on the first two consecutive days in ICU.

**Results:**

Overall, 20 (44.4%) patients required insulin to achieve target blood glucose. Insulin-treated patients had higher glycated hemoglobin A1c, more premorbid insulin-requiring type 2 diabetes, and greater blood glucose levels but lower c-peptide levels on admission. Premorbid insulin-requiring diabetes was independently associated with lower admission c-peptide, whereas greater plasma creatinine was independently associated with higher levels. Increases in c-peptide were positively correlated with an increase in blood glucose both in patients who did (*r* = 0.54, *P* = 0.01) and did not (*r* = 0.56, *P* = 0.004) receive insulin. However, insulin administration was independently associated with a greater increase in c-peptide (*P* = 0.04). This association was not modified by the use of oral insulin secretagogues.

**Conclusions:**

C-peptide, a marker of beta-cell response, responds to and is influenced by glycemia and renal function in critically ill patients with type 2 diabetes. In addition, in our cohort, exogenous insulin administration was associated with a greater increase in c-peptide in response to hyperglycemia.

*Trial Registration* Australian New Zealand Clinical Trials Registry (ACTRN12615000216516).

**Electronic supplementary material:**

The online version of this article (doi:10.1186/s13613-017-0274-5) contains supplementary material, which is available to authorized users.

## Background

Stress-induced hyperglycemia is common in critically ill patients due to acute metabolic alterations leading to increased hepatic glucose release and reduced glucose uptake in peripheral tissues [1, 2]. In patients with preserved beta-cell function, increased endogenous insulin secretion counter-balances such insulin resistance.

In contrast, in patients with type 2 diabetes mellitus (T2DM), endogenous insulin secretion may be insufficient to maintain glucose homeostasis during additional, stress-induced insulin resistance as occurs during critical illness. In such patients, exogenous insulin administration is often required to control blood glucose levels within a target range.

However, continuous intravenous insulin infusion does not adequately replicate the physiological response and effects of endogenous insulin secretion. Endogenous insulin is secreted in a pulsatile fashion into the portal vein where the amplitude of these oscillations appears associated with both hepatic and extra-hepatic insulin delivery and insulin-receptor sensitivity [3]. Furthermore, preserved endogenous insulin secretion prevents potentially harmful iatrogenic hypoglycemia and the increased glucose variability associated with insulin therapy [4]. Finally, insulin is co-secreted with c-peptide, a biologically active amino acid polypeptide cleaved from the proinsulin molecule of pancreatic beta-cells. Data suggest that c-peptide improves insulin sensitivity and attenuates the long-term complications associated with type 1 diabetes [5]. In addition, c-peptide may ameliorate the systemic inflammatory response associated with critical illness [6]. Hence, supporting such physiological beta-cell function during acute illness may be a desirable physiological and clinical goal. In this regard, recent data suggest that insulin administration may support glucose-stimulated endogenous insulin secretion in insulin-sensitive and, to a lesser degree, insulin-resistant subjects [7]. In contrast, in a cohort of 339 critically ill patients (11% with a history of diabetes) with stress-hyperglycemia and markedly elevated c-peptide levels, intensive insulin therapy targeting normoglycemia reduced endogenous insulin secretion and normalized c-peptide levels [8]. However, information about whether insulin therapy stimulates or suppresses the beta-cell response during permissive, stress-induced hyperglycemia in critically ill T2DM patients has not been studied.

Accordingly, we conducted a prospective, observational, exploratory study to assess the effect of insulin therapy on early levels of c-peptide as a surrogate marker of beta-cell function during moderate permissive hyperglycemia in patients with T2DM admitted to ICU. We hypothesized that the magnitude of such hyperglycemia would be associated with the level of circulating c-peptide levels in patients with T2DM. Furthermore, we hypothesized that insulin administration would be associated with a greater increase in c-peptide levels in response to hyperglycemia.

## Methods

The study was approved by the ethics committee at Austin Hospital, Melbourne, Australia (approval number LNR/14/Austin/487), with a waiver for informed consent.

### Patient selection and data collection

We prospectively included consecutive adult (≥18 years) patients with known T2DM admitted to a single tertiary ICU from November 14, 2015 to May 18, 2016. According to our local unit protocol for diabetic patients [9, 10], intravenous or subcutaneous insulin was administered at a blood glucose level (BGL) above 14 mmol/l targeting a BGL between 10 and 14 mmol/l. Diabetes diagnosis was confirmed by the medical records or by the next-of-kin. Patients with a hyperglycemic hyperosmolar state or diabetic ketoacidosis were excluded.

We recorded the following baseline variables: age, sex, body weight, diabetes therapy prior to ICU admission [insulin, oral hypoglycemic agents (OHA), diet], admission glycated hemoglobin A1c (HbA1c), source of and reason for ICU admission, and Acute Physiology and Chronic Health Evaluation (APACHE) III score. Intravenous (IV) and subcutaneous (SC) units of insulin and type and dose of OHAs administrated in the 24 before and initial 24 h following ICU admission were also recorded. Additionally, we recorded the total caloric intake via enteral and/or parenteral routes during this time frame. Study patients were categorized into two groups based on whether they received insulin (insulin group) or not (non-insulin group) during the initial 24 h in ICU. We also compared patients who did and did not receive oral insulin secretagogues (sulfonylureas and/or dipeptidyl peptidase-4 inhibitors) at any time in the 24 before and/or initial 24 h following ICU admission.

### Blood and urine sampling and measurements

Plasma c-peptide and creatinine were measured in routine blood samples obtained on ICU admission (ICU day 1) and on the morning of the following day in ICU (ICU day 2). Arterial blood gas and urine samples obtained at corresponding time-points were used to measure blood glucose levels, blood ketone levels [β-hydroxybutyrate (β-OHB)] and urine ketones. Plasma c-peptide was analyzed at the hospital laboratory using the Cobas e 602 analyzer (Roche Diagnostics, Mannheim, Germany). The normal fasting reference range for c-peptide reported by the laboratory is 0.33–1.47 nmol/l.

We measured blood β-OHB using the Freestyle Optium Xceed point-of-care meter (Abbott Diabetes Care Inc., UK). Ketonuria was semi-quantified using Combur-test^®^ strips (Roche Diagnostics, Rotkreuz, Switzerland). We defined ketonemia as a blood β-OHB level ≥0.6 mmol/l. Ketonuria was defined as a urine ketone level ≥1 mmol/l. Arterial blood glucose was analyzed using the Radiometer ABL825 blood gas analyzer (Radiometer Medical A/S, Brønshøj, Denmark). HbA1c was analyzed using COBAS INTEGRA 800 (Roche, Rotkreuz, Switzerland) [11].

### Statistical analysis

We analyzed data using STATA^®^ version Stata/SE 11.2 (Stata Corp., College Station, TX, USA). Continuous variables were expressed as median (IQR), and categorical variables as *n* (%). We compared continuous data using the Mann–Whitney *U* test and categorical data using the Chi-square test or the Fisher’s exact test. Correlations were assessed using Spearman’s rank. We used multivariable linear regression analysis to explore the independent association between admission c-peptide level and the following variables: Insulin-requiring diabetes (yes vs. no), insulin administration within 24 h before admission c-peptide measurement (yes vs. no and as a continuos variable in units), admission blood glucose level, HbA1c level, creatinine level and APACHE III score. To determine the effect of insulin administration on beta-cell function and endogenous insulin secretion during 24 h of moderate permissive hyperglycemia in ICU, we used multivariable linear regression analysis (using the percentage change in c-peptide level between ICU day 1 and 2 as dependent variable) adjusting for insulin-requiring diabetes (yes vs. no), percentage change in blood glucose, admission HbA1c level, admission creatinine level, APACHE III score and administration of insulin secretagogues (yes vs. no). In the regression analyses, we used backward selection of variables with a *P* value <0.1. A two-sided *P* value <0.05 was considered statistically significant in the final analyses.

## Results

### Patient characteristics and outcomes

We studied a convenience sample of 45 consecutive T2DM patients (44.4% females) with a median (IQR) age of 68 (61, 77) years, APACHE III score of 64 (43, 76) and a HbA1c of 6.7 (6.2, 7.3)% (Table 1). Details of administration of insulin and oral hypoglycemic agents to these patients are found in Table 2 and in Additional file 1: Table S1. Eight (17.8%) patients received insulin secretagogues in the 24 before and/or initial 24 h following ICU admission. Overall, 20 (44.4%) patients received insulin (insulin group) between ICU day 1 and 2 (median total dose 24 [14, 46] units) (Table 1).

**Table 1:** Baseline characteristics and outcomes for all patients and according to insulin therapy during the first 24 h in ICU

Characteristic	All patients (*n* = 45)	Non-insulin group (*n* = 25)	Insulin group (*n* = 20)	*P*
Age (years)	68 (61, 77)	69 (60, 78)	67 (64, 73)	0.53
Female sex, *n* (%)	20 (44.4%)	14 (56%)	6 (30%)	0.08
APACHE III score	64 (43, 76)	65 (48, 86)	64 (42, 72)	0.42
HbA1c (%)	6.7 (6.2, 7.3)	6.4 (5.9, 6.9)	7.1 (6.3, 7.8)	0.02
Body weight (kg)	80 (72, 93)	75 (71, 85)	87 (73, 97)	0.14
Chronic insulin therapy, *n* (%)	12 (26.7%)	4 (16.0%)	8 (40.0%)	0.07
Source of ICU admission, *n* (%)				
Operating theater	20 (44.4%)	9 (36%)	11 (55%)	0.55
Emergency Department	7 (15.6%)	5 (20%)	2 (10%)	
Ward	11 (24.4%)	6 (24%)	5 (25%)	
Other hospital	7 (15.6%)	5 (20%)	2 (10%)	
Vasopressor therapy on admission, *n* (%)	15 (33.3%)	6 (24%)	9 (45%)	0.14
Mechanical ventilation on admission, *n* (%)	21 (46.7%)	10 (40%)	11 (55%)	0.32
ICU length of stay (days)	2.6 (1.7, 3.9)	2.6 (1.8, 3.9)	2.7 (1.4, 4.6)	1.0
Hospital length of stay (days)	10 (6.8, 15.0)	9.6 (4.9, 15.0)	11 (8.0, 18.0)	0.27
ICU mortality, *n* (%)	4 (8.9%)	4 (16%)	0	–
Hospital mortality, *n* (%)	4 (8.9%)	4 (16%)	0	–

Values are median (IQR) or *n* (%)

**Table 2:** Biochemical variables, glycemic therapy and nutrition

Variable	All patients (*n* = 45)	Non-insulin group (*n* = 25)	Insulin group (*n* = 20)	*P*
Blood glucose level (mmol/l)				
Day 1	9.9 (7.0, 11.0)	7.9 (6.4, 10.0)	11.0 (9.8, 12.0)	<0.001
Day 2	9.8 (8.1, 12.0)	8.7 (6.8, 11.0)	12.0 (9.7, 16.0)	<0.001
Plasma C-peptide level (nmol/l)				
Day 1	1.3 (0.8, 2.9)	1.6 (0.9, 2.3)	0.9 (0.3, 3.0)	0.19
Day 2	1.6 (0.9, 2.3)	1.7 (1.3, 2.1)	1.3 (0.6, 3.4)	0.29
Creatinine level (µmol/l)				
Day 1	127 (73, 195)	105 (68, 195)	138 (91, 189)	0.55
Day 2	113 (73, 216)	92 (61, 216)	117 (90, 222)	0.49
Ketonemia, *n* (%)^a^				
Day 1	12/34 (35.3%)	6/18 (33.3%)	6/16 (37.5%)	0.80
Day 2	11/36 (30.6%)	8/19 (42.1%)	3/17 (17.6%)	0.16
Ketonuria, *n* (%)^b^				
Day 1	3/34 (8.8%)	0/18	3/16 (18.8%)	–
Day 2	3/36 (8.3%)	2/19 (10.5%)	1/17 (5.9%)	1.0
Insulin therapy, *n* (%)				
Before day 1	13 (28.9%)	4 (16.0%)	9 (45.0%)	0.049
Between day 1 and day 2	20 (44.4%)	0	20 (100%)	–
Total dose in treated, units	–	–	24 (14, 46)	–
Any insulin secretagogue^c^				
Before day 1	3 (7)	1 (4)	2 (10)	0.58
Between day 1 and day 2	7 (16)	2 (8)	5 (25)	0.21
Enteral nutrition, *n* (%)^d^				
Day 1	2 (4.4%)	1 (4.0%)	0	–
Day 2	9 (20.0%)	6 (24.0%)	3 (15.0%)	0.71
Parenteral nutrition, *n* (%)^d^				
Day 1	1 (2.2%)	0	1 (5.0%)	–
Day 2	2 (4.4%)	1 (4.0%)	1 (5.0%)	1.0

Values are median (IQR) or *n* (%)

^a^Blood ketone level ≥0.6 mmol/l

^b^Urine ketone level ≥1 mmol/l

^c^Sulfonylureas and/or dipeptidyl peptidase-4 inhibitors

^d^Ongoing nutrition at the time of c-peptide measurement

### C-peptide levels, kidney function, ketosis and glycemic control

Compared to non-insulin group patients, insulin group patients had a significantly greater median BGL on day 1 (11.0 [9.8, 12.0] vs. 7.9 [6.4, 10.0] mmol/l, *P* < 0.001) and on day 2 (12.0 [9.7, 16.0] vs. 8.7 [6.8, 11.0] mmol/l, *P* < 0.001). However, c-peptide levels did not differ significantly between the groups on either day.

Creatinine levels and the proportion of patients with ketonemia and/or ketonuria did not differ significantly between the groups. Overall, 9 (45.0%) and 4 (16.0%) patients received insulin within 24 h before ICU admission in the insulin and non-insulin group, respectively (*P* = 0.049). Insulin secretagogues were administered to a greater proportion of patients in the insulin group. Parenteral or enteral nutrition was delivered to less than a quarter of patients (Table 2).

Although not reaching statistical significance, we observed slightly higher c-peptide on day 2 in patients receiving insulin secretagogues (*n* = 8) than in patients not receiving such therapy (*n* = 37). A greater proportion of patients receiving insulin secretagogues had elevated c-peptide. A greater proportion of these patients also received insulin (Additional file 1: Table S2).

### Association with beta-cell function on ICU admission

On multivariable linear regression analysis, the presence of premorbid insulin-requiring diabetes was independently associated with lower admission c-peptide level (−0.9 nmol/l, 95% CI −1.8 to −0.04, *P* = 0.04). Furthermore, higher admission creatinine level was independently associated with higher c-peptide level. Insulin administration within 24 h before admission c-peptide measurement, admission BGL, HbA1c and APACHE III score were not retained in the model (Table 3).

**Table 3:** Multivariable linear regression analysis of the association with admission c-peptide levels (nmol/l)

Variable	Univariable analysis		Multivariable analysis	
	Crude estimate (95% CI)	*P* value	Adjusted estimate^a^ (95% CI)	*P* value
Insulin-requiring diabetes				
No	Reference		Reference	
Yes	−0.9 (−1.9 to 0.09)	0.07	−0.9 (−1.8 to −0.04)	0.04
Creatinine level, per 10 µmol/l	0.05 (0.02 to 0.08)	0.003	0.05 (0.02 to 0.08)	0.002

^a^Insulin administration within 24 h before admission c-peptide measurement, admission blood glucose level, HbA1c and APACHE III score were not retained in the model. Backward selection was used to include variables with *P* < 0.1 in the final model

### Insulin therapy and beta-cell response during persistent hyperglycemia

**Fig. 1:**
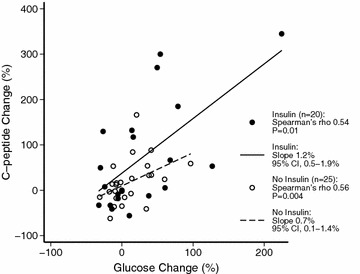
*Scatterplot with fitted regression lines* showing the relationship between the change in c-peptide and blood glucose during the first 24 h in ICU among patients who did (*closed circles*, *solid line*) and did not (*open circles*, *dashed line*) receive insulin

The percentage change in c-peptide between ICU day 1 and 2 positively correlated with the corresponding percentage change in BGL in patients who did (Spearman’s rho 0.54, *P* = 0.01) and did not (Spearman’s rho 0.56, *P* = 0.004) receive insulin during this time frame. However, in patients receiving insulin, c-peptide increased by 1.2% (95% CI 0.5–1.9%) per percentage increase in BGL. In patients not receiving insulin, c-peptide increased by 0.7% (95% CI 0.1–1.4%) per percentage increase in BGL (Fig. 1). On univariable linear regression analysis, insulin administration was associated with a 55.2% (95% CI 2.0–108.4%) greater increase in c-peptide than no insulin administration (*P* = 0.04). On multivariable regression analysis adjusting for the presence of premorbid insulin-requiring diabetes and BGL change, insulin administration was independently associated with a greater percentage increase in c-peptide than no insulin administration (*P* = 0.04) (Table 4). In addition, a greater insulin dose was independently associated with a greater percentage c-peptide increase (Additional file 1: Table S3). HbA1c level, creatinine level, APACHE III score and administration of insulin secretagogues were not retained in the models.

**Table 4:** Multivariable linear regression analysis of the association with c-peptide change (%) from ICU admission to the next-day value

Variable	Univariable analysis		Multivariable analysis	
	Crude estimate (95% CI)	*P* value	Adjusted estimate^b^ (95% CI)	*P* value
Insulin administration^a^				
No	Reference		Reference	
Yes	55.2 (2.0 to 108.4)	0.04	45.9 (2.8 to 89.0)	0.04
Insulin-requiring diabetes				
No	Reference		Reference	
Yes	7.0 (−55.7 to 69.8)	0.82	−59.7 (−111.2 to −8.2)	0.02
Blood glucose change, per %	1.2 (0.7 to 1.6)	<0.001	1.3 (0.8 to 1.8)	<0.001

^a^Intravenous and/or subcutaneous insulin administered between ICU day 1 and ICU day 2

^b^HbA1c level, creatinine level, APACHE III score and administration of insulin secretagogues were not retained in the model. Backward selection was used to include variables with *P* < 0.1 in the final model

## Discussion

### Key findings

In a prospective observational study, we measured c-peptide as a biomarker of endogenous insulin secretion during moderate permissive hyperglycemia in critically ill adult patients with T2DM. Premorbid insulin-requiring diabetes was associated with lower levels of c-peptide and higher plasma creatinine with greater levels of c-peptide. Although, as expected, c-peptide increased in response to a corresponding increase in BGL, early insulin therapy (median 24 units over 24 h) augmented such glucose-stimulated c-peptide secretion independent of the presence of premorbid insulin-requirement, or magnitude of change in BGL. Additionally, a higher insulin dose was associated with a greater c-peptide increase. Finally, we observed somewhat greater c-peptide in patients receiving insulin secretagogues. However, such therapy did not significantly modify the relationship between insulin administration and beta-cell response.

### Relationship to previous studies

To date, no study has evaluated early c-peptide secretion during permissive hyperglycemia in critically ill T2DM patients. However, Langouche et al. compared insulin and c-peptide levels between critically ill patients with stress-hyperglycemia randomized to intensive insulin therapy (target BGL 4.4–6.1 mmol/l) or conventional glucose control (target BGL 10–11.1 mmol/l), of whom the majority (approximately 90%) did not have a history of diabetes [8]. Compared with a cohort of 26 fasted healthy volunteers, c-peptide levels were higher in critically ill patients on ICU admission. Moreover, compared with our cohort of T2DM patients, c-peptide levels on admission and on day 2 were higher in the conventional-control patients in the study by Langouche et al. despite lower blood glucose and similar amount of administered insulin (20–25 units per 24 h). This suggests that our T2DM patients had some degree of beta-cell insufficiency. Furthermore, whereas c-peptide remained elevated in their conventional-control patients during the first week in ICU, c-peptide normalized after achieving normoglycemia in the intensive insulin therapy group. This observation supports our finding that blood glucose change is a major determinant of c-peptide secretion.

The effect of insulin on beta-cell function was previously investigated in volunteers. In healthy volunteers, exogenous insulin administration suppressed c-peptide release during normoglycemia [12, 13]. This suppressive effect was, however, attenuated during mild hyperglycemia in subjects with non-insulin dependent diabetes [14]. In contrast, Anderwald et al. found increased c-peptide secretion during exposure to hyperinsulinemic normoglycemia in healthy subjects, whereas c-peptide levels decreased during insulin infusion and persistent normoglycemia in subjects with impaired glucose tolerance or established T2DM [15].

As these volunteer studies were conducted during strict normoglycemia, their relevance for critically ill T2DM patients with permissive stress-hyperglycemia is limited. More relevant is a recent study measuring endogenous insulin and c-peptide levels during graded hyperglycemia (dextrose infusion to a maximum BGL of 18 mmol/l) following a 4-h isoglycemic clamp with either saline (no insulin, sham) or insulin (2 milliunits/kg/min) in healthy volunteers and in subjects with T2DM [7]. Pre-exposure to low-dose insulin enhanced hyperglycemia-induced endogenous insulin and c-peptide secretion in both groups. This effect was, however, attenuated in subjects with T2DM. These findings support the notion that exogenous insulin, possibly via autocrine effects, stimulates insulin secretion and that this positive feedback-mechanism is suppressed in patients with stressed beta-cells, such as T2DM patients [16]. However, in our cohort of patients with acute-on-chronic insulin resistance, we observed a greater percentage increase in c-peptide levels in insulin-treated patients than in patients not receiving insulin during hyperglycemia. This effect persisted after adjusting for the presence of premorbid insulin-requiring diabetes and for the magnitude change in BGL. Although an autocrine insulin effect could potentially explain our finding, we cannot rule out that attenuated glucotoxicity (decreased BGL) during insulin infusion contributed to improved beta-cell function [17].

In addition to hyperglycemia, impaired kidney function is known to increase plasma c-peptide levels as approximately half of circulating c-peptide is cleared via the kidneys [18]. Our data, showing an independent association between plasma creatinine and c-peptide levels on admission, support this notion.

Emerging data suggest that c-peptide is not an inactive by-product of insulin secretion but may in fact play an important role during systemic inflammatory stress. For example, treatment with c-peptide after the induction of endotoxic shock in mice attenuated the systemic inflammatory response and improved survival compared with vehicle [6]. In addition, treatment with c-peptide during resuscitation for hemorrhagic shock in rats ameliorated hypotension, blunted the systemic inflammatory response and reduced neutrophil infiltration in the lung tissue [19]. Whether c-peptide has similar beneficial effects in critically ill humans is yet to be determined. However, other studies suggest that an elevated c-peptide level is independently associated with micro- and macrovascular complications in T2DM patients [20]. Accumulation of c-peptide in atherogenic plaques and chemotactic effects of c-peptide on monocytes may be involved in the pathophysiology of such complications [21].

### Implications of study findings

Our findings imply that hyperglycemia stimulates endogenous c-peptide (and therefore insulin) secretion even in critically ill patients with acute-on-chronic insulin resistance. Furthermore, they imply that early exogenous insulin therapy has no suppressive effect on the ability of beta-cells to secrete c-peptide (and therefore insulin) during stress-hyperglycemia. On the contrary, in our cohort, the independent association between insulin therapy and an increase in c-peptide during hyperglycemia supports a previous hypothesis [7] that exogenous insulin may stimulate beta-cell function (beta-cell recruitment). Such recruitment would logically help deliver more insulin into the portal vein and restore a more physiological splanchnic effect of insulin. Moreover, our findings imply that kidney function needs to be considered when interpreting c-peptide levels. We failed to demonstrate a significant association between insulin secretagogue therapy and beta-cell function in our study cohort. However, only eight patients received such therapy and, consequently, we acknowledge that a lack of effect could be a type 2 error. The effect of insulin sensitizers and secretagogues on beta-cell function and glycemic control needs to be explored in future trials.

### Study strengths and limitations

Our study has several strengths. To our knowledge, we are the first to explore early c-peptide levels in a heterogeneous cohort of type 2 diabetic patients with critical illness. Second, we collected detailed information on premorbid diabetic treatment and glycemic control, acute glycemic control, illness severity, and acute kidney function and were therefore able to assess the independent relationship between these variables and c-peptide levels. Third, we analyzed c-peptide levels over two consecutive days in the same laboratory using the same platform to understand changes in response to intervention. Fourth, clinicians were blinded to the c-peptide results, which allowed us to make an unbiased assessment of the relationship between c-peptide and insulin administration in ICU. Finally, our diabetic patients were studied during moderate permissive hyperglycemia, which has two important implications. The first is that it has the potential to trigger greater insulin release allowing us to more clearly assess beta-cell functional reserve. The second is that our liberal glucose target reduced the proportion of patients requiring exogenous insulin therapy, which allowed us assess the impact of insulin therapy in these patients. Yet, we acknowledge that permissive hyperglycemia is not standard practice in most centers. The observed effect of insulin on c-peptide may be less pronounced or even absent in patients receiving a “tighter” glucose control protocol.

Our study also has several limitations. It is a single-center study, decreasing the generalizability of our findings to other centers. However, our ICU has all the typical features of an academic ICU within a tertiary hospital in a developed country, suggesting a degree of external validity. We did not measure insulin levels. However, c-peptide levels more accurately reflect endogenous insulin production during insulin therapy [22]. Yet, we believe that both c-peptide and insulin levels should be reported in future studies to better understand the physiology of beta-cells under these circumstances. We only assessed c-peptide levels during the first 2 days in ICU. Therefore, we cannot draw any conclusions about longer-term beta-cell function during persistent hyperglycemia in ICU. The presence of an incretin effect induced by enteral nutrition, an important physiological trigger of beta-cell stimulation [23], was not explored. However, this effect was likely negligible in our cohorts; only one patient in the non-insulin group and no patients in the insulin group received enteral nutrition on day 1. We did not use a reference method, such as a normoglycemic hyperinsulinemic clamp technique, to quantify insulin resistance. However, such clamp techniques are not feasible in the rapidly changing ICU environment and may in themselves affect endogenous insulin secretion. We did not include healthy controls or critically ill patients without diabetes. However, permissive hyperglycemia in nondiabetic patients would represent a significant deviation from our unit’s practice and was therefore not possible. Finally, a proportion of our patients that had received early insulin therapy had significantly greater BGLs during the study period than patients not requiring insulin. This may have enhanced the c-peptide response in the insulin group. However, our findings persisted after adjusting for blood glucose or blood glucose change in multivariable analyses.

## Conclusions

In this cohort of critically ill patients with type 2 diabetes, insulin therapy was associated with enhanced secretion of c-peptide in response to stress-induced hyperglycemia. The effect of oral hypoglycemic agents on beta-cell response and glycemic control need further investigations.

## Additional file

**Additional file 1: Table S1.** Details of insulin administration and use of oral hypoglycemic agents. **Table S2**. Biochemical variables and insulin therapy in patients who did and did not receive secretagogues in the 24 h before ICU admission and/or during the first 24 h in ICU. **Table S3**. Multivariable linear regression analysis of the association with c-peptide change (%) from ICU to the next-day value.

## Abbreviations

APACHE: Acute Physiology and Chronic Health Evaluation
β-OHB: β-hydroxybutyrate
BGL: blood glucose level
HbA1c: glycated hemoglobin A1c
ICU: intensive care unit
IV: intravenous
IQR: interquartile range
OHA: oral hypoglycemic agents
SC: subcutaneous
T2DM: type 2 diabetes mellitus
