# Supplementary material for: The effect of insulin administration on c-peptide in critically ill patients with type 2 diabetes
Source: Ann Intensive Care. 2017 May 12;7:50. doi: 10.1186/s13613-017-0274-5 (PMC5427062; doi:10.1186/s13613-017-0274-5)
Supplement: Supplementary file 1 — Additional file 1: Table S1. Details of insulin administration and use of oral hypoglycemic agents. Table S2. Biochemical variables and insulin therapy in patients who did and did not receive secretagogues in the 24 h before ICU admission and/or during the first 24 h in ICU. Table S3. Multivariable linear regression analysis of the association with c-peptide change (%) from ICU to the next-day value. [file 13613_2017_274_MOESM1_ESM.docx]

Additional file 1

**The Effect of Insulin Administration on C-Peptide in Critically Ill Patients with Type 2 Diabetes**

**Table S1**. Details of insulin administration and use of oral hypoglycemic agents.

| **Variable** | **All patients**  **(n = 45)** | **Non-insulin group**  **(n = 25)** | **Insulin**  **group**  **(n = 20)** | **P*** |
| --- | --- | --- | --- | --- |
| Intravenous insulin therapy |  |  |  |  |
| Before Day 1 | 12 (27) | 3 (12) | 9 (45) | 0.02 |
| Between Day 1 and Day 2 | 14 (31) | 0 | 14 (70) | <0.001 |
| Subcutaneous insulin therapy |  |  |  |  |
| Before Day 1 | 3 (7) | 1 (4) | 2 (10) | 0.56 |
| Between Day 1 and Day 2 | 7 (16) | 0 | 7 (35) | 0.002 |
| Biguanides |  |  |  |  |
| Before Day 1 | 5 (11) | 4 (16) | 1 (5) | 0.36 |
| Between Day 1 and Day 2 | 1 (2) | 1 (4) | 0 | 1.0 |
| Sulfonylureas |  |  |  |  |
| Before Day 1 | 1 (2) | 0 | 1 (5) | 0.44 |
| Between Day 1 and Day 2 | 4 (9) | 0 | 4 (20) | 0.03 |
| Dipeptidyl Peptidase-4 Inhibitors |  |  |  |  |
| Before Day 1 | 2 (4) | 1 (4) | 1 (5) | 1.0 |
| Between Day 1 and Day 2 | 4 (9) | 2 (8) | 2 (10) | 1.0 |
| Any oral hypoglycemic agent, n (%) |  |  |  |  |
| Before Day 1 | 7 (15.6) | 4 (16.0) | 3 (15.0) | 1.0 |
| Between Day 1 and Day 2 | 8 (17.8) | 3 (12.0) | 5 (25.0) | 0.44 |
| Values are n (%)  *Fisher's exact test | | | | |

**Table S2**. Biochemical variables and insulin therapy in patients who did and did not receive secretagogues in the 24 hours before ICU admission and/or during the first 24 hours in ICU.

| **Variable** | **No Secretagogue group**  **(n = 37)** | **Secretagogue group**  **(n = 8)** | **P** |
| --- | --- | --- | --- |
| Blood Glucose Level, mmol/l |  |  |  |
| Day 1 | 9.4 (7.0, 11.0) | 10.0 (7.6, 11.0) | 0.67 |
| Day 2 | 11.0 (8.6, 12.0) | 8.9 (6.8, 14.0) | 0.56 |
| Plasma C-peptide Level, nmol/l |  |  |  |
| Day 1 | 1.3 (0.6, 2.7) | 1.5 (0.9, 3.0) | 0.81 |
| Day 2 | 1.6 (0.9, 4.4) | 1.9 (1.2, 3.1) | 0.66 |
| Creatinine Level, µmol/l |  |  |  |
| Day 1 | 120 (72, 212) | 138 (94, 177) | 0.77 |
| Day 2 | 109 (72, 216) | 123 (90, 215) | 0.70 |
| Ketonemia, n (%)^a^ |  |  |  |
| Day 1 | 10/28 (36) | 2/6 (33) | 1.0 |
| Day 2 | 11/29 (38) | 0 | 0.08 |
| Ketonuria, n (%)^b^ |  |  |  |
| Day 1 | 1/28 (4) | 2/6 (33) | 0.07 |
| Day 2 | 2/29 (7) | 1/7 (14) | 0.49 |
| Insulin therapy, n (%) |  |  |  |
| Before Day 1 | 10 (27) | 3 (38) | 0.67 |
| Between Day 1 and Day 2 | 14 (38) | 6 (75) | 0.11 |
| Values are median (IQR) or n (%)  ^a^Blood ketone level ≥0.6 mmol/l  ^b^Urine ketone level ≥1 mmol/l | | | |

**Table S3**. Multivariable linear regression analysis of the association with c-peptide change (%) from ICU to the next day value.

|  | **Univariable analysis** | | **Multivariable analysis** | |
| --- | --- | --- | --- | --- |
| **Variable** | **Crude Estimate**  **(95% CI)** |  | **Adjusted Estimate**  **(95% CI)** | **P-value** |
| Insulin administration, per unit^a^ | 0.6 (-0.5 to 1.5) | 0.29 | 0.9 (0.04 to 1.8) | 0.04 |
| Insulin-requiring diabetes |  |  |  |  |
| No |  |  | Reference |  |
| Yes | 7.0 (-55.7 to 69.8) | 0.82 | -72.2 (-127.7 to -16.8) | 0.01 |
| Blood glucose change, per % | 1.2 (0.7 to 1.6) | <0.001 | 1.4 (0.9 to 1.9) | <0.001 |
| ^a^Total intravenous and subcutaneous insulin dose administered between ICU day 1 and ICU day 2. | | | | |
